# Design and Practical Considerations for Active Polymeric Films in Food Packaging

**DOI:** 10.3390/ijms23116295

**Published:** 2022-06-04

**Authors:** Wing-Fu Lai, Wing-Tak Wong

**Affiliations:** 1 School of Life and Health Sciences, The Chinese University of Hong Kong (Shenzhen), Shenzhen 518172, China; 2 Department of Applied Biology and Chemical Technology, Hong Kong Polytechnic University, Hong Kong SAR, China; w.t.wong@polyu.edu.hk

**Keywords:** food packaging, polymers, active agents, food preservation, packaging films

## Abstract

Polymeric films for active food packaging have been playing an important role in food preservation due to favorable properties including high structural flexibility and high property tunability. Over the years, different polymeric active packaging films have been developed. Many of them have found real applications in food production. This article reviews, using a practical perspective, the principles of designing polymeric active packaging films. Different factors to be considered during materials selection and film generation are delineated. Practical considerations for the use of the generated polymeric films in active food packaging are also discussed. It is hoped that this article cannot only present a snapshot of latest advances in the design and optimization of polymeric active food packaging films, but insights into film development to achieve more effective active food packaging can be attained for future research.

## 1. Introduction

Packaging plays an important role in extending the shelf life of food products and retarding product deterioration [1,2]. Most of the food products sold in the market are put inside packages, which can provide barrier effects to protect the products from microbial contamination and environmental attacks (such as oxygen, water vapor, and light). Among different packaging materials, polymers have been widely adopted. This is largely because, compared to other packaging materials (such as wood and glass), polymers have greater flexibility and versatility in their structures and functions [3,4,5,6,7,8,9]. Their properties can be more easily tuned to meet the needs of different food packaging applications [10,11]. In fact, not only can packages made of polymers be easily generated in different dimensions and shapes, many of them are also thermosealable and microwavable [12,13,14,15]. In addition, they can be transparent. This allows the appearance of the product inside to be seen by consumers and is appealing in the product design perspective [16,17]. Along with their lightness, low cost, and ease of printing, polymers have been serving as an important class of materials in food packaging [18].

Despites the promising potential as mentioned above, packages generated from polymers are highly permeable to gases and other low-molecular-weight compounds. This reduces the effectiveness of these food packages to serve as a passive barrier to combat the adverse effects caused by the surrounding to the packaged food. To tackle this problem, one strategy is to control the atmosphere inside the food package by putting a sachet of chemicals (e.g., ethylene scavengers, carbon dioxide emitters, oxygen scavengers, and humidity adsorbers) inside the package [18]. This approach greatly enhances the efficiency of the polymer-generated package to extend the shelf life of the food product even without the use of additional complex technologies and film modification [18]. However, the chemicals used inside the sachet are often non-consumable. Accidental release of these agents from the sachet to the food causes safety concern [19,20,21,22]. An alternative to this approach is the incorporation of active agents into polymer-generated packages. This approach of active packaging, in which the package interacts actively with the environment and/or the food during food preservation, has drawn extensive interest over the last several decades [18]. Until now, different polymer-generated active food packaging films have been developed [23,24,25,26,27]. While multiple articles have already been published to review the latest advances in the development of these films [28,29,30,31,32,33], efforts devoted to presenting the current understanding of the process of film design are scant. This review fills this gap by revisiting various major factors and strategies known to be important for an active food packaging film to be properly designed, produced, and optimized. It is anticipated that this review will help lay a theoretical and practical foundation for further development of more effective films for active food packaging.

## 2. Design of Film Compositions for Food Packaging

**Figure 1:**
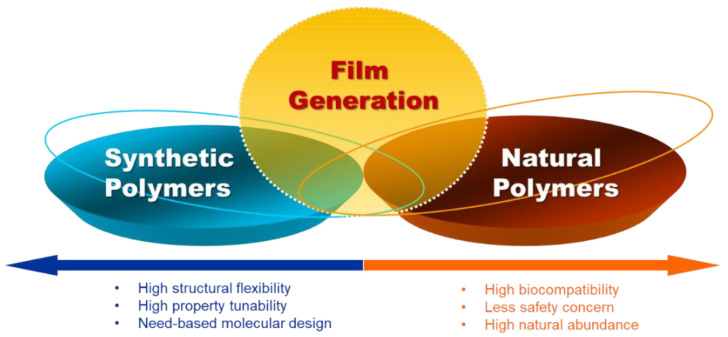
Types and features of polymers used for generation of active food packaging films.

Selection of polymers is pivotal when a packaging film is designed. One major group of polymers that have been widely exploited in film fabrication is natural polymers, who show natural abundance and high sustainability (Figure 1). They can help reduce the use of petroleum resources, and reduce problems led by environmental pollution. Natural polymers adopted for film generation include chitosan [34,35], starch [36], and cellulose [37]. Recently, fruit purées have been exploited for film making. For instance, edible films containing cinnamaldehyde nanoemulsions have been generated from pectin and papaya purée and have been reported to show antibacterial properties against *Staphylococcus aureus*, *Salmonella enterica*, *Escherichia coli*, and *Listeria monocytogenes* [38]. More recently, a biodegradable and edible packaging film has been developed by using papaya purée. During film fabrication, papaya purée is added to distilled water containing starch, followed by heating at 75 °C for 30 min to obtain a film-forming solution. Upon the addition of glycerol as a plasticizer, a film is generated via solution casting [39]. To enhance the performance of the film in food packaging, gelatin and defatted soy protein have been incorporated. Such incorporation has been shown to lead to an enhancement in elongation at break and a decrease in water solubility [39].

An alternative to natural polymers are synthetic polymers [40,41,42,43,44]. One example is polyethylene (PE), which has been adopted in food packaging since the 1940s [45]. Its wide application is partially attributed to its low cost, recyclability, and easy feedstock availability. The role played by PE in food packaging has recently been further enhanced by the possible attainment of ethylene feedstock from renewable resources, thereby enabling the generation of PE from renewal natural sources with properties identical to petroleum-derived counterparts [46]. Among different types of PE, low-density polyethylene (LDPE) has been extensively adopted for fabrication of food packaging films due to its stiffness, moisture barrier capacity, and high transparency. Owing to its thermal stability, it can be used in melt-flow processes [47]. The use of LDPE in food packaging has been exemplified by the case of the biocomposite film consisting of LDPE and curcumin [48]. The film has been generated by melt extrusion and hot pressing for active food packaging [48]. Besides PE, other synthetic polymers used in food packaging include poly(lactic acid) (PLA), poly(ε-caprolactone) (PCL) [49], and poly(vinyl alcohol) (PVA) [50]. For instance, an antioxidant packaging film has recently been generated from PVA upon crosslinking with critic acid (CA) and the incorporation of bioactive ingredients [50].

Apart from using either natural or synthetic polymers, films combining both types of polymers have been generated. These films show higher tunability and flexibility than those generated from either type of polymers. This is demonstrated by the antibacterial film generated from polyethylenimine (PEI) and soy protein isolate (SPI). During the fabrication process, SPI and PEI are mixed and heated under constant stirring. The heating process leads to denaturation of SPI, causing destruction of the α-helical conformation and unfolding of the random coil [51]. Moreover, an increase in the kinetic energy of the SPI molecule causes rearrangement of the chain structure and the formation of β-pleated sheets [51]. This structural change is further facilitated by the presence of PEI, which can interrupt the chain of the protein and destroy the crystalline structure, resulting in a further increase in amorphicity [51]. All these result in better mixing of the two polymers in the aqueous environment, and, importantly, more effective formation of hydrogen bonds between PEI and SPI [51]. By incorporating different structural polymers into a film-forming solution, it is expected that the stiffness and toughness of the resulting film can be better tuned [52]. Owing partly to its highly branched structure and short chain length, the PEI moiety renders the generated SPI/PEI film ductile and stretchable [51]. On the other hand, the comparatively high crystallinity of the SPI moiety can increase the mechanical strength of the generated film [51]. The mechanical strength of the SPI/PEI film has been further improved by the presence of metal ions. This is attributed to the fact that addition of metal ions leads to the formation of metal-ligand interactions [51], which replace the hydrogen bonds formed between SPI and PEI and lead to an increase in the tensile strength of the film.

## 3. Strategies for the Production of Polymeric Films

Over the years, various methods have been applied to generate packaging films, including solution casting, electrospinning, and melt extrusion. The choice of the method, along with different process parameters, can significantly alter the properties of the generated film and, hence, the performance in active food packaging.

### 3.1. Solution Casting

**Figure 2:**
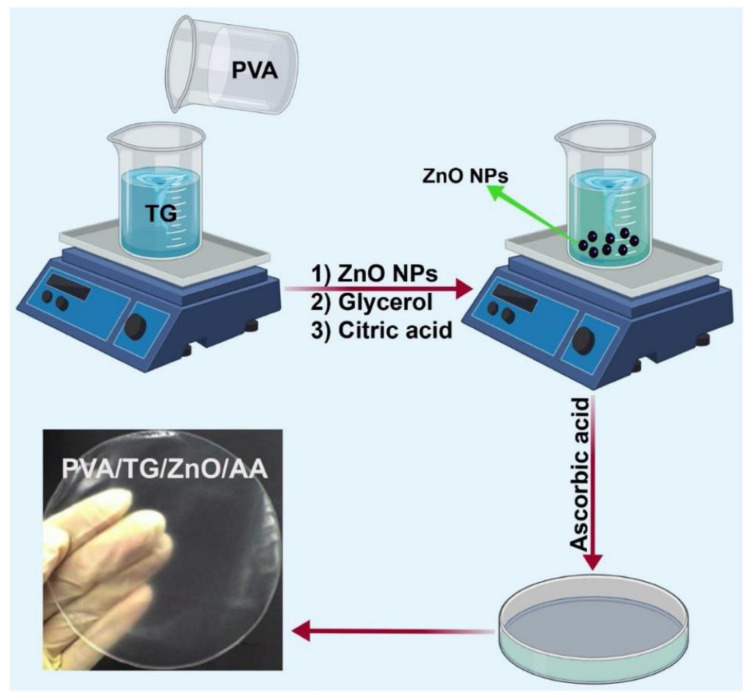
A schematic diagram showing the fabrication of an TG-based nanocomposite film. Adapted with permission from [50], 2020, Elsevier B.V.

Solution casting, in which a film-forming solution containing the active ingredient is poured onto a flat surface, followed by evaporation of the solvent, is one strategy of fabricating polymeric films for food packaging. The use of this method has been demonstrated by the fabrication of bioactive nanocomposite films composed of tragacanth (TG), PVA, ZnO nanoparticles, and ascorbic acid (AA) [50]. During film preparation, an aqueous solution of PVA is added to an aqueous solution of TG, followed by the addition of dispersed ZnO nanoparticles. Afterwards, glycerol (which serves as a plasticizer) and CA (which serves as a cross-linker) are added. The solution mixture is poured into a petri dish after the addition of AA to form cross-linked films that show antioxidant properties (Figure 2) [50]. In fact, solution casting is the most extensively used method in the literature for the production of food packaging films. This is attributed to its low cost as well as its ease of operation. Furthermore, only simple pieces of apparatus (e.g., plastic dish and glass plate) are needed during film production, therefore, it can be performed basically in any laboratory. Despite this, one major drawback of solution casting is the difficulty of ensuring batch-to-batch consistency in the properties of the generated films because the performance of the films is subjected to influences of the time and temperature of drying. In addition, large-scale production is difficult when solution casting is adopted.

### 3.2. Melt Extrusion

**Figure 3:**
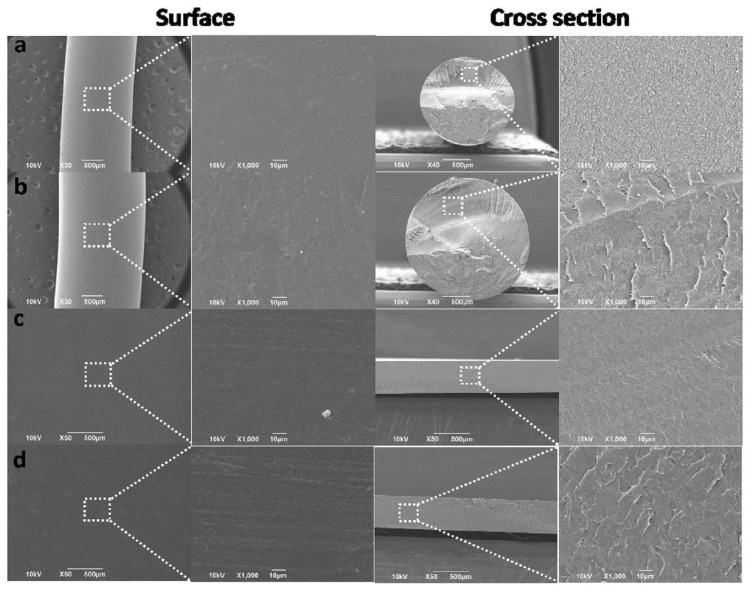
Surface and cross-sectional morphologies of the (**a**) pure LDPE filament, (**b**) curcumin-loaded LDPE filament, (**c**) pure LDPE film, and (**d**) curcumin-loaded LDPE film. Adapted with permission from [48], 2019, Elsevier B.V.

The use of melt extrusion in generating food packaging films is exemplified by the case of the biocomposite film comprising LDPE and curcumin [48]. During the process, curcumin and LDPE are first coextruded into filaments, which are subsequently transformed into pellets. An active food packaging film is generated upon hot pressing of the pellets. After the incorporation of curcumin into the LDPE filaments and the film, there is a slight reduction in smoothness of the surface morphology; however, the appearance of air gaps, big particle agglomerates, detachment zones, or cracks has not been observed, indicating the good dispersion of curcumin in the polymer matrix (Figure 3) [48]. More recently, polypropylene (PP)-based films incorporated with synthetic antioxidants have been generated via melt exclusion, in which a mixture of PP granules and antioxidants is first transferred to a co-rotating twin-screw extruder with the barrel temperature at 145–200 °C [53]. The molten extrudate then leaves the die in a string form and is cut into granules upon passing through a cold-water basin. The granules finally are added into another single-screw blowing extruder to generate film strips.

### 3.3. Electrospinning

Electrospinning is a technique of fiber fabrication. It deposits an electrically-charged single jet on a grounded collector, enabling the production of fibrous non-woven materials whose surface-area-to-volume ratio is much higher than that of the film counterparts. Due to the high surface area-to-volume-ratio, the antioxidant present in the fiber can be more accessible to radicals formed during oxidation of the food product, thereby enhancing the efficiency in reducing or controlling the rate of food oxidation. The possible use of electrospinning in film preparation for active food packaging is demonstrated by an earlier study [54], in which lentil flour/poly(ethylene oxide) (PEO) nanofibers loaded with gallic acid have been generated by electrospinning as an active food packaging material. The fibers have been found to display strong antioxidant capacity. Walnuts packed in nanofibers have been shown to have smaller amounts of peroxides and hydroperoxides formed during the initial stage of lipid oxidation and have a lower total oxidation (TOTOX) value [54]. This demonstrates the ability of the package generated by using the nanofibers to enhance the oxidative stability of the walnuts. More recently, a composite film has been generated by using the electrospinning technology for food preservation, too [55]. During film fabrication, PLA is first dissolved in trichloromethane, followed by the addition of MgO and ZnO. A film is formed from the resultant solution via solution casting. After that, a solution containing gelatin and eugenol was deposited on the surface of the PLA-based film using an electrospinning device to generate a double-layer composite film. Finally, the composite film is annealed by being placed between two Teflon layers and compressed at 70 °C for 1 min, generating a film with antioxidant capacity for food packaging.

**Figure 4:**
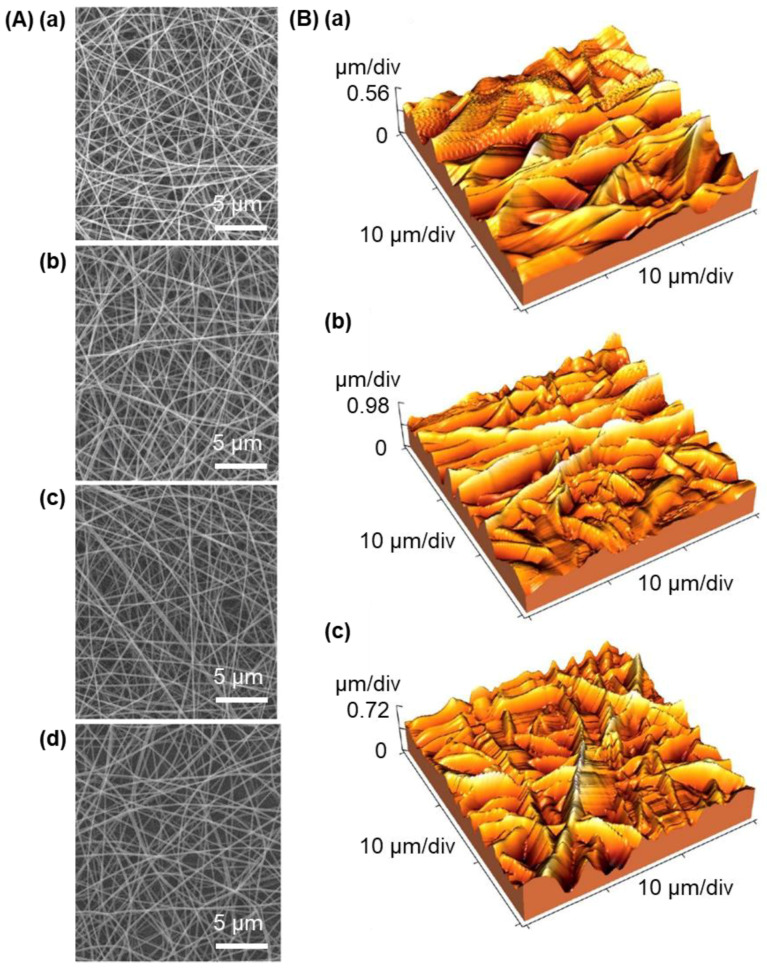
(**A**) Scanning electron microscopy images of electrospun nanofiber mats consisting of chitosan and PVA and being loaded with different concentrations of protein hydrolysates: (**a**) 0 mg/mL, (**b**) 1 mg/mL, (**c**) 3 mg/mL, and (**d**) 5 mg/mL. (**B**) 3D atomic force microscopy images of electrospun nanofiber mats consisting of (**a**) PVA alone, (**b**) PVA and chitosan, and (**c**) chitosan, PVA as well as protein hydrolysates. Adapted with permission from [56], 2019, Elsevier B.V.

During electrospinning, the rate of vaporization of the solvent from the surface of the jet has to be properly controlled. A rapid rate is needed for the generation of fibers with the tube-like morphology [55]. On the other hand, a non-uniform rate may lead to fiber adhesion [55]. Apart from this, the concentration of the active agent added to the spinning solution may change the viscosity, causing droplets more difficult to be split and, hence, a larger diameter of the nanofibers. This has been demonstrated by Hosseini and co-workers [56], who have generated an electrospun nanofibrous mat consisting of chitosan and PVA. In the mat, fish-derived protein hydrolysates obtained from *Clupeonella cultriventris* caspia have been adopted as the antioxidant agent. An increase in the concentration of the protein hydrolysates has been found to lead to an increase in the fiber diameter (Figure 4). Meanwhile, due to an increase in the viscosity of the spinning solution upon the addition of chitosan and protein hydrolysates, comparing with the electrospun fibrous mat generated from PVA alone, those containing chitosan and protein hydrolysates have been found to have a higher value of root mean square (RMS) roughness for their surface.

## 4. Optimization of Film Properties for Food Packaging

Not only are the properties of a generated active packaging film affected by the film composition and by the fabrication approach, but the conditions adopted for film generation can significantly change the performance of a packaging film. One good example is the drying condition adopted during the preparation of a film made by solution casting. Variations in the drying condition lead to changes in the ultimate film properties. This is demonstrated by an earlier study, which has examined the effect of different drying protocols (including bench casting at 28–32 °C for 12–48 h, drying in a dehydrator at 50 °C for 4 h, or drying in a convection oven at 138 °C for 5 min) on properties of papaya edible films [57]. The one dried in a convection oven has been found to have the highest b* value (indicating that the color of the film has the highest degree of yellowness), and this is attributed to the occurrence of non-enzymatic browning. Owing to caramelization of sugars, a distinct caramel-like odor is detected in the oven-dried film [57]. The thickness and water activity of the film have also been found to be affected by the drying protocol adopted, with the film generated by bench casting showing the highest water activity and film thickness [57]. All these demonstrate the importance of proper design of procedures for film fabrication in order to attain high-quality food packaging films.

Apart from taking the design and fabrication of a film *per se* into consideration, active packaging films generally contain active ingredients. The impact of these ingredients on properties of the generated film as a whole should not be overlooked. In the following parts of this section, the concentration of active agents and the nature of film components will be further discussed for optimization of active food packaging films.

### 4.1. Concentrations of an Active Ingredient

The addition of an active ingredient to the polymer matrix of a film may lead to changes in film properties, such as mechanical strength, wettability, and vapor permeability. This has been revealed partly by the case of the PVA/TG/ZnO/AA bionanocomposite film, in which the amounts of TG and ZnO nanoparticles have been found to be negatively related to the aqueous solubility of the film [50]. Moreover, in the case of the curcumin-containing LDPE film, an increase in the weight percentage of curcumin has been shown to lead to an increase in the antioxidant properties as demonstrated by the 2,2-diphenyl-1-picrylhydrazyl (DPPH) assay [48]; however, the rigidity of the film as well as the elongation at breakage decline [48], resulting in a reduction in the performance of the film in food packaging as a whole. This reveals the need of properly characterizing changes in film properties during film development to ensure the efficiency of the packaging film in serving as a barrier is not compromised by the presence of the active ingredient.

The importance of taking active agents into consideration during film development has also been revealed recently by the case of the antibacterial self-healable PEI/SPI film [51]. Incorporation of different metal ions into the film has been found to significantly change the self-healing properties, with the self-healing capacity of the copper ion-incorporated SPI/PEI film being much higher than that of the zinc ion-incorporated counterpart [51]. Such a difference in self-healing properties is attributed to the difference in the second ionization energy of the metal ions. Compared with zinc ions, copper ions have higher second ionization energy [58]. This allows Cu(II) ions to be more effective in accepting the electron pair from the nitrogen atoms of PEI, leading to more effective formation of coordination bonds during the self-healing process. Besides the selection of the metal ions, proper control of the metal ion content appears to be needed for optimal film performance [51]. This is demonstrated by the observation that an increase in the metal ion content leads to a decrease in the stretchability of the SPI/PEI film [51]. Such a phenomenon can be partly explained by the significant reduction in the mobility of polymeric chains when too many hydrogen bonds are replaced by coordination bonds. Due to the presence of dynamic reversible non-covalent interactions in the metal ion-incorporated SPI/PEI film, the film shows good self-healing properties [51]. Compared with the plain SPI/PEI film, the one containing metal ions has been found to be more effective to mediate self-healing [51]. All these evidence the impact of active agents on the properties of packaging films, and call for attention during the process of film design and optimization.

In addition, optical properties of the film may be changed upon the incorporation of the active ingredient. Opacity is one important parameter of film appearance which can influence the degree of consumer acceptance. The color of a film can also affect the potential of the film in food packaging due to its effect on general appearance and consumer acceptance of the packaged food product [59]. The effect of the incorporation of an active ingredient on the optical properties of a packaging film has been revealed by the case of the khorasan wheat starch film containing the leaf extract of *Moringa oleifera* L. [36]. An increase in the concentration of the extract in the film leads to changes in film color from yellow green to dark green, with a decrease in the L* value and an increase in opacity.

### 4.2. Nature and Loading of an Active Ingredient

The nature of an active ingredient is a factor determining the applicability and commercial value of the film design. This is particularly true when the plant extract is adopted as an active ingredient during film fabrication because the performance of the film may be affected by the season and production location of the plant. This has been demonstrated by Iqbal and Bhanger [60], who studied the antioxidant activity of the methanolic extract of *Moringa oleifera* leaves harvested in different seasons and agroclimatic locations. They found that the overall antioxidant activity is higher in the extract of leaves harvested in December or March, depending on the location, but is the least when harvested in June [60]. Meanwhile, samples from Mardaan have been shown to have the highest antioxidant activity, followed by Balakot, Chakwal, Jamshoro, and Nawabshah [60]. Understanding this during film development is of practical importance because it implies that when packaging films incorporated with plant extracts are developed and produced in the industrial context, one area that has to be taken into consideration is the feasibility of obtaining a constant supply of the target plant whose bioactivity can be maintained, or the performance of the films in food protection will fluctuate from batch to batch.

To load an active ingredient into a film, various strategies can be adopted. One commonly used method is plasma treatment, which can modify the properties of only an extremely thin surface layer of a material without affecting the bulk properties. The use of plasma treatment for chemically loading active substances onto the film surface has been exemplified by the case reported by Potrc and colleagues [61], who first increased the surface free energy (as well as the bonding ability) of a polymer film by treating the film with O_2_ plasma, followed by the addition of chitosan nanoparticles, in which the pomegranate extract or catechin had been loaded, to the film. Not only can O_2_ plasma generate binding sites to facilitate the adherence of chitosan nanoparticles to the film surface, it can also inhibit desorption of chitosan. In comparison with the untreated film, the film that has undergone plasma treatment has exhibited much higher antioxidant activity. Apart from plasma treatment, ultraviolet irradiation is a feasible method for modification of film surface to enable agent loading. The feasibility of this has been demonstrated by Muriel-Galet and coworkers [62], who adopted ultraviolet irradiation to generate arboxylic acid groups on the surface of poly(vinyl alcohol-*co*-ethylene) films. Increasing the treatment time with UV leads to an increase in the amount of carboxylic acid groups generated. Those groups enable covalent immobilization of active substances on the film surface.

## 5. Practical Considerations for Applications in Active Food Packaging

**Figure 5:**
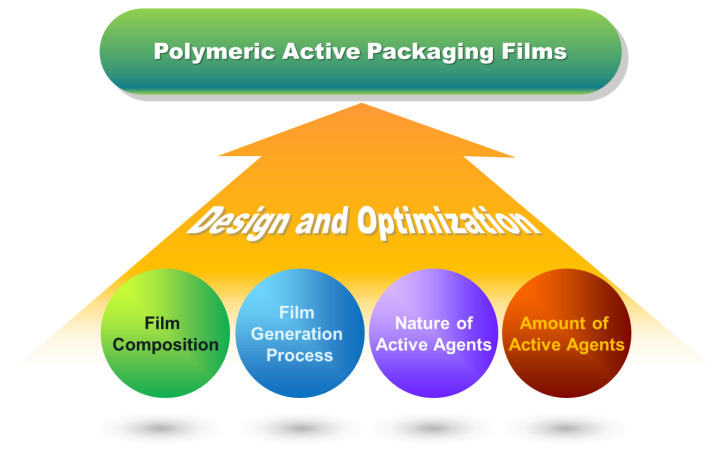
Major factors to be considered during the design of polymeric active food packaging films.

Proper selection of the film-forming materials, active agents, and the conditions for film production is pivotal when maximizing the effect of a generated film in keeping the quality of the food product (Figure 5). In fact, while optimizing the physical properties of the film is required if effective use in food preservation is to be achieved, many other factors have to be practically considered before a film can be applied to the production line. One of these factors is the mechanism of action of the active agent incorporated into the film. If the active ingredient in the film is expected to migrate to the food product for action, that ingredient should be approved to be used as a food additive. In addition, the amount of that ingredient in the food should be tightly regulated by present regulations, and should not be too high that the sensory attributes of the food are affected.

Apart from the mechanism of action in active food packaging, variations in the performance of the film under different environmental conditions should be considered. This is because the environmental conditions, in which the film is used and the packaged food is stored, will vary greatly among different food stores and manufacturers. For this, even if a film is demonstrated to be effective, its effectiveness may be different when it is practically used. The impact of environmental factors to the performance of a film has been demonstrated previously by the case of the antibacterial SPI-PEI film [51], in which an increase in temperature can increase the mobility of molecular chains and can enhance the restoration of hydrogen bonds and coordination interactions in the film, thereby altering the self-healing process and the restoration time [51]. All these demonstrate the need of taking the environmental impact on the properties and performance of a film into account when the film is applied to package a food product.

If an active agent incorporated into a film is not supposed to be transferred to a food product, possible undesired migration of the agent into the food product from the food packaging film should be seriously examined. Over the years, a number of migration models have been proposed to assess migration of additives and contaminants from a packaging film into a food product [63]. Below are two of the commonly adopted models that are developed based on Fick’s Second Law [63], where *L_P_* is the thickness of the packaging film, *D* is the diffusion coefficient of the migrant in the packaging film, *M_F,t_* is the amount of the migrant in the packaged food at time *t*, *M_F,__∞_* is the amount of the migrant in the packaged food at equilibrium, and *M*_*P,*0_ is the initial amount of the migrant in the packaging film.

(1) $$\frac{M_{F,t}}{M_{F,\infty }}=\frac{2}{L_{P}}{\left(\frac{Dt}{\pi }\right)}^{0.5}$$

(2) $$\frac{M_{F,t}}{M_{P,0}}=\frac{2}{L_{P}}{\left(\frac{Dt}{\pi }\right)}^{0.5}$$

Although the models can effectively estimate the diffusion coefficient when partitioning and resistance to mass transfer are insignificant [63], they can hardly accurately be used to determine the diffusion coefficient for partitioned migration [63]. Even though other migration models have been proposed [64,65,66,67], each of these models has specific limitations in providing a strong correlation between theoretical and practical observation under different conditions. Development of effective models and characterization methods to evaluate food-packaging interactions will be one important area that plays a vital role in facilitating the design and optimization of safe and effective polymeric films for active food packaging. Last but not least, when a food packaging film is proposed to be translated from the laboratory into the industry, the cost and scalability of film production, as well as the marketability of the film, have to be considered. These factors can directly affect the possible success of the translation process.

## 6. Environmental Sustainability of Active Food Packaging

While the barrier properties and packaging performance of a film predominately determine the application prospects, the environmental impact of the film should not be overlooked. In fact, packaging waste has been a subject of environmental concern over the years. It occupies around 65% of waste volume because of its bulkiness [68]. Packaging waste includes the used or scrap packaging materials as well as the solid waste produced during process operations. Recycling of packaging waste is particularly challenging when different polymers co-exist either as blends or as copolymers in food packages [68]. Right now, different strategies (including source reduction, composting, recycling, and incineration) have been exploited as alternatives to landfilling packaging waste [69]; however, in order to more effectively combat the environmental issue caused by packaging waste, efforts have to be paid during the design and production of the food packaging film. In particular, biodegradable polymeric films are preferred over non-degradable synthetic plastic films [70,71,72,73]. A cradle-to-grave life-cycle assessment should also be performed to assess the overall environmental burden brought about by the production, application and disposal of a food packaging film [69]. This enables more effective management and prediction of the environmental impact caused by the film at the design stage.

In fact, right now, active food packaging has already been practically used in various countries, including Japan, Australia, and the US [74]. However, regulations governing the use of active food packaging are scant [74]. In addition, although lots of studies related to the development of polymeric active food packaging films have been reported in the literature, some of those studies have focused only on the structural and physical characterization of the generated films without having the films tested on real food products [75]. These, along with other technical problems and limitations (e.g., failure of a migration model for accurate estimation of food-packaging interactions) are some of the problems that have to be addressed in the forthcoming decades so as to facilitate the translation of more polymeric active food packaging films from the laboratory context to food industry.

## 7. Concluding Remarks and Outlooks

Development of active food packaging films, as compared to the use of sachets for active food packaging, enables a reduction in the package size and an increase in the effectiveness of food protection (due to the fact that active substances can more effectively surround the food product). For this, it is expected that active food packaging films will play an increasingly important role in food preservation in the coming future. Clearly lots of technical problems (including the cost, scalability of film production, marketability, and environmental impact), as mentioned above, have to be addressed in order to streamline the application of the generated films in active food packaging. In addition, different types of food products have specific mechanisms of spoilage. One film may not necessarily be applicable to all food products. Future research to enhance the understanding of the spoilage mechanism of different food types is needed to provide more insights into the development of more effective films for food preservation. Nevertheless, regarding the promising potential of active food packaging and the gradual sophistication of technologies for film production, polymeric active packaging films are anticipated to continue playing an important role in food production in the upcoming decades.

## Data Availability

Not applicable.

## References

[1] Parhi A., Tang J.M., Sablani S.S. (2020). Functionality of ultra-high barrier metal oxide-coated polymer films for in-package, thermally sterilized food products. Food Packag. Shelf Life.

[2] de Carvalho A.P.A., Conte C.A. (2020). Green strategies for active food packagings: A systematic review on active properties of graphene-based nanomaterials and biodegradable polymers. Trends Food Sci. Technol..

[3] Hu K.Q., Huyan Z.Y., Ding S.X., Dong Y.Y., Yu X.Z. (2020). Investigation on food packaging polymers: Effects on vegetable oil oxidation. Food Chem..

[4] Fernandes S.S., Romani V.P., Filipini G.D., Martins V.G. (2020). Chia seeds to develop new biodegradable polymers for food packaging: Properties and biodegradability. Polym. Eng. Sci..

[5] Diez-Pascual A.M. (2020). Antimicrobial polymer-based materials for food packaging applications. Polymers.

[6] Lai W.F., Huang E.M., Wong W.T. (2020). A gel-forming clusteroluminogenic polymer with tunable emission behavior as a sustained-release carrier enabling real-time tracking during bioactive agent delivery. Appl. Mater. Today.

[7] Lai W.F. (2020). Non-conjugated polymers with intrinsic luminescence for drug delivery. J. Drug Deliv. Sci. Technol..

[8] Li T., Shang D., Gao S., Wang B., Kong H., Yang G., Shu W., Xu P., Wei G. (2022). Two-dimensional material-based electrochemical sensors/biosensors for food safety and biomolecular detection. Biosensors.

[9] Li X., Yue X., Huang Q., Zhang B. (2022). Effects of wet-media milling on multi-scale structures and in vitro digestion of tapioca starch and the structure-digestion relationship. Carbohydr. Polym..

[10] Tran T.N., Mai B.T., Setti C., Athanassiou A. (2020). Transparent bioplastic derived from CO_2_-based polymer functionalized with oregano waste extract toward active food packaging. ACS Appl. Mater. Int..

[11] Makvandi P., Iftekhar S., Pizzetti F., Zarepour A., Zare E.N., Ashrafizadeh M., Agarwal T., Padil V.V.T., Mohammadinejad R., Sillanpaa M. (2021). Functionalization of polymers and nanomaterials for water treatment, food packaging, textile and biomedical applications: A review. Environ. Chem. Lett..

[12] Topuz F., Uyar T. (2020). Antioxidant, antibacterial and antifungal electrospun nanofibers for food packaging applications. Food Res. Int..

[13] Nesic A., Cabrera-Barjas G., Dimitrijevic-Brankovic S., Davidovic S., Radovanovic N., Delattre C. (2019). Prospect of polysaccharide-based materials as advanced food packaging. Molecules.

[14] Huang T., Qian Y., Wei J., Zhou C. (2019). Polymeric antimicrobial food packaging and its applications. Polymers.

[15] Chen X., Chen M., Xu C., Yam K.L. (2019). Critical review of controlled release packaging to improve food safety and quality. Crit. Rev. Food Sci. Nutr..

[16] Lai W.F., Zhao S., Chiou J. (2021). Antibacterial and clusteroluminogenic hypromellose-graft-chitosan-based polyelectrolyte complex films with high functional flexibility for food packaging. Carbohydr. Polym..

[17] Lai W.F., Wong W.T. (2022). Edible clusteroluminogenic films obtained from starch of different botanical origins for food packaging and quality management of frozen foods. Membranes.

[18] Lai W.F. (2021). Design of polymeric films for antioxidant active food packaging. Int. J. Mol. Sci..

[19] Velazquez-Contreras F., Zamora-Ledezma C., Lopez-Gonzalez I., Meseguer-Olmo L., Nunez-Delicado E., Gabaldon J.A. (2021). Cyclodextrins in polymer-based active food packaging: A fresh look at nontoxic, biodegradable, and sustainable technology trends. Polymers.

[20] Roy S., Priyadarshi R., Ezati P., Rhim J.W. (2022). Curcumin and its uses in active and smart food packaging applications—A comprehensive review. Food Chem..

[21] Ozdemir M., Floros J.D. (2004). Active food packaging technologies. Crit. Rev. Food Sci. Nutr..

[22] Basavegowda N., Baek K.H. (2021). Advances in Functional Biopolymer-Based Nanocomposites for Active Food Packaging Applications. Polymers.

[23] Zaitoon A., Luo X., Lim L.T. (2022). Triggered and controlled release of active gaseous/volatile compounds for active packaging applications of agri-food products: A review. Compr. Rev. Food Sci. Food Saf..

[24] Sultana A., Kathuria A., Gaikwad K.K. (2022). Metal-organic frameworks for active food packaging. A review. Environ. Chem. Lett..

[25] Raul P.K., Thakuria A., Das B., Devi R.R., Tiwari G., Yellappa C., Kamboj D.V. (2022). Carbon nanostructures as antibacterials and active food-packaging materials: A review. ACS Omega.

[26] Ludwicka K., Kaczmarek M., Bialkowska A. (2020). Bacterial nanocellulose—A biobased polymer for active and intelligent food packaging applications: Recent advances and developments. Polymers.

[27] Krasniewska K., Galus S., Gniewosz M. (2020). Biopolymers-based materials containing silver nanoparticles as active packaging for food applications—A review. Int. J. Mol. Sci..

[28] Mahmud J., Sarmast E., Shankar S., Lacroix M. (2022). Advantages of nanotechnology developments in active food packaging. Food Res. Int..

[29] Hamed I., Jakobsen A.N., Lerfall J. (2022). Sustainable edible packaging systems based on active compounds from food processing byproducts: A review. Compr. Rev. Food Sci. Food Saf..

[30] Casalini S., Giacinti Baschetti M. (2022). The use of essential oils in chitosan or cellulose-based materials for the production of active food packaging solutions: A review. J. Sci. Food Agric..

[31] Vianna T.C., Marinho C.O., Marangoni Junior L., Ibrahim S.A., Vieira R.P. (2021). Essential oils as additives in active starch-based food packaging films: A review. Int. J. Biol. Macromol..

[32] Soltani Firouz M., Mohi-Alden K., Omid M. (2021). A critical review on intelligent and active packaging in the food industry: Research and development. Food Res. Int..

[33] Sharma S., Barkauskaite S., Jaiswal A.K., Jaiswal S. (2021). Essential oils as additives in active food packaging. Food Chem..

[34] Kadam A.A., Singh S., Gaikwad K.K. (2021). Chitosan based antioxidant films incorporated with pine needles (*Cedrus deodara*) extract for active food packaging applications. Food Control..

[35] Rambabu K., Bharath G., Banat F., Show P.L., Cocoletzi H.H. (2019). Mango leaf extract incorporated chitosan antioxidant film for active food packaging. Int. J. Biol. Macromol..

[36] Ju A., Baek S.K., Kim S., Song K.B. (2019). Development of an antioxidative packaging film based on khorasan wheat starch containing moringa leaf extract. Food Sci. Biotechnol..

[37] Bastante C.C., Silva N.H.C.S., Cardoso L.C., Serrano C.M., Martínez de la Ossa E.J., Freire C.S.R., Vilela C. (2021). Biobased films of nanocellulose and mango leaf extract for active food packaging: Supercritical impregnation versus solvent casting. Food Hydrocoll..

[38] Otoni C.G., Moura M.R.D., Aouada F.A., Camilloto G.P., Cruz R.S., Lorevice M.V., Soares N.d.F.F., Mattoso L.H.C. (2014). Antimicrobial and physical-mechanical properties of pectin/papaya puree/cinnamaldehyde nanoemulsion edible composite films. Food Hydrocoll..

[39] Tulamandi S., Rangarajan V., Rizvi S.S.H., Singhal R.S., Chattopadhyay S.K., Saha N.C. (2016). A biodegradable and edible packaging film based on papaya puree, gelatin, and defatted soy protein. Food Packag. Shelf Life.

[40] Lai W.F. (2022). Non-aromatic clusteroluminogenic polymers: Structural design and applications in bioactive agent delivery. Mater. Today Chem..

[41] Lai W.F., Deng R., He T.C., Wong W.T. (2021). A bioinspired, sustained-release material in response to internal signals for biphasic chemical sensing in wound therapy. Adv. Healthc. Mater..

[42] Lai W.F., Hu C.S., Deng G.X., Lui K.H., Wang X., Tsoi T.H., Wang S.X., Wong W.T. (2019). A biocompatible and easy-to-make polyelectrolyte dressing with tunable drug delivery properties for wound care. Int. J. Pharmaceut..

[43] Lai W.F., Wong W.T. (2018). Design of polymeric gene carriers for effective intracellular delivery. Trends Biotechnol..

[44] Lai W.F., Rogach A.L. (2017). Hydrogel-based materials for delivery of herbal medicines. ACS Appl. Mater. Int..

[45] Risch S.J. (2009). Food packaging history and innovations. J. Agric. Food Chem..

[46] Bos H.L., Meesters K.P.H., Conijn S.G., Corre W.J., Patel M.K. (2012). Accounting for the constrained availability of land: A comparison of bio-based ethanol, polyethylene, and PLA with regard to non-renewable energy use and land use. Biofuels Bioprod. Biorefin..

[47] Siracusa V., Blanco I. (2020). Bio-polyethylene (Bio-PE), bio-polypropylene (Bio-PP) and bio-poly(ethylene terephthalate) (Bio-PET): Recent developments in bio-based polymers analogous to petroleum-derived ones for packaging and engineering applications. Polymers.

[48] Zia J., Paul U.C., Heredia-Guerrero J.A., Athanassiou A., Fragouli D. (2019). Low-density polyethylene/curcumin melt extruded composites with enhanced water vapor barrier and antioxidant properties for active food packaging. Polymer.

[49] Lukic I., Vulic J., Ivanovic J. (2020). Antioxidant activity of PLA/PCL films loaded with thymol and/or carvacrol using scCO2 for active food packaging. Food Packag. Shelf Life.

[50] Janani N., Zare E.N., Salimi F., Makvandi P. (2020). Antibacterial tragacanth gum-based nanocomposite films carrying ascorbic acid antioxidant for bioactive food packaging. Carbohyd. Polym..

[51] Li F., Ye Q., Gao Q., Chen H., Shi S.Q., Zhou W., Li X., Xia C., Li J. (2019). Facile fabrication of self-healable and antibacterial soy protein-based films with high mechanical strength. ACS Appl. Mater. Interfaces.

[52] Yang Y., Wang X., Yang F., Wang L., Wu D. (2018). Highly elastic and ultratough hybrid ionic-covalent hydrogels with tunable structures and mechanics. Adv. Mater..

[53] Fasihnia S.H., Peighambardoust S.H., Peighambardoust S.J., Oromiehie A., Soltanzadeh M., Peressini D. (2020). Migration analysis, antioxidant, and mechanical characterization of polypropylene-based active food packaging films loaded with BHA, BHT, and TBHQ. J. Food Sci..

[54] Aydogdu A., Yildiz E., Aydogdu Y., Sumnu G., Sahin S., Ayhan Z. (2019). Enhancing oxidative stability of walnuts by using gallic acid loaded lentil flour based electrospun nanofibers as active packaging material. Food Hydrocoll..

[55] Li M., Yu H., Xie Y., Guo Y., Cheng Y., Qian H., Yao W. (2021). Fabrication of eugenol loaded gelatin nanofibers by electrospinning technique as active packaging material. LWT.

[56] Hosseini S.F., Nahvi Z., Zandi M. (2019). Antioxidant peptide-loaded electrospun chitosan/poly(vinyl alcohol) nanofibrous mat intended for food biopackaging purposes. Food Hydrocoll..

[57] Rodríguez G.M., Sibaja J.C., Espitia P.J.P., Otoni C.G. (2020). Antioxidant active packaging based on papaya edible films incorporated with Moringa oleifera and ascorbic acid for food preservation. Food Hydrocoll..

[58] Gao B.J., An F.Q., Liu K.K. (2006). Studies on chelating adsorption properties of novel composite material polyethyleneimine/silica gel for heavy-metal ions. Appl. Surf. Sci..

[59] Srinivasa P.C., Ramesh M.N., Kumar K.R., Tharanathan R.N. (2003). Properties and sorption studies of chitosan-polyvinyl alcohol blend films. Carbohyd. Polym..

[60] Iqbal S., Bhanger M.I. (2006). Effect of season and production location on antioxidant activity of Moringa oleifera leaves grown in Pakistan. J. Food Compos. Anal..

[61] Potrc S., Krasevac Glaser T., Vesel A., Poklar Ulrih N., Fras Zemljic L. (2020). Two-layer functional coatings of chitosan particles with embedded catechin and pomegranate extracts for potential active packaging. Polymers.

[62] Muriel-Galet V., Talbert J.N., Hernandez-Munoz P., Gavara R., Goddard J.M. (2013). Covalent immobilization of lysozyme on ethylene vinyl alcohol films for nonmigrating antimicrobial packaging applications. J. Agric. Food Chem..

[63] Chung D., Papadakis S.E., Yam K.L. (2002). Simple models for assessing migration from food-packaging films. Food Addit. Contam..

[64] Stoffers N.H., Dekker M., Linssen J.P.H., Stormer A., Franz R., van Boekel M.A.J.S. (2005). Modelling of simultaneous two-sided migration into water and olive oil from nylon food packaging. Eur. Food Res. Technol..

[65] Pocas M.F., Oliveira J.C., Brandsch R., Hogg T. (2012). Analysis of mathematical models to describe the migration of additives from packaging plastics to foods. J. Food Process. Eng..

[66] Gavriil G., Kanavouras A., Coutelieris F.A. (2018). Food-packaging migration models: A critical discussion. Crit. Rev. Food Sci..

[67] Douziech M., Benitez-Lopez A., Ernstoff A., Askham C., Hendriks A.J., King H., Huijbregts M.A.J. (2020). A regression-based model to predict chemical migration from packaging to food. J. Expo. Sci. Environ. Epidemiol..

[68] Arvanitoyannis I.S., Bosnea L.A. (2001). Recycling of polymeric materials used for food packaging: Current status and perspectives. Food Rev. Int..

[69] Bohlmann G.M. (2004). Biodegradable packaging life-cycle assessment. Environ. Prog..

[70] Islamipour Z., Zare E.N., Salimi F., Ghomi M., Makvandi P. (2022). Biodegradable antibacterial and antioxidant nanocomposite films based on dextrin for bioactive food packaging. J. Nano Struct. Chem..

[71] Cvek M., Paul U.C., Zia J., Mancini G., Sedlarik V., Athanassiou A. (2022). Biodegradable films of PLA/PPC and curcumin as packaging materials and smart indicators of food spoilage. ACS Appl. Mater. Int..

[72] Akshaykranth A., Jayarambabu N., Rao T.V., Kumar R.R., Rao L.S. (2022). Antibacterial activity study of ZnO incorporated biodegradable poly (lactic acid) films for food packaging applications. Polym. Bull..

[73] Ahmed M., Verma A.K., Patel R. (2022). Physiochemical, antioxidant, and food simulant release properties of collagen-carboxymethyl cellulose films enriched with Berberis lyceum root extract for biodegradable active food packaging. J. Food Process. Preserv..

[74] Lopez-Rubio A., Almenar E., Hernandez-Munoz P., Lagaron J.M., Catala R., Gavara R. (2004). Overview of active polymer-based packaging technologies for food applications. Food Rev. Int..

[75] Ahvenainen R., Hurme E. (1997). Active and smart packaging for meeting consumer demands for quality and safety. Food Addit. Contam..

